# Neuroimaging biomarkers to associate obesity and negative emotions

**DOI:** 10.1038/s41598-017-08272-8

**Published:** 2017-08-09

**Authors:** Bo-yong Park, Jisu Hong, Hyunjin Park

**Affiliations:** 1 Department of Electronic, Electrical and Computer Engineering, Sungkyunkwan University, Suwon, Korea; 2 Center for Neuroscience Imaging Research, Institute for Basic Science (IBS), Suwon, Korea; 3 School of Electronic and Electrical Engineering, Sungkyunkwan University, Suwon, Korea

## Abstract

Obesity is a serious medical condition highly associated with health problems such as diabetes, hypertension, and stroke. Obesity is highly associated with negative emotional states, but the relationship between obesity and emotional states in terms of neuroimaging has not been fully explored. We obtained 196 emotion task functional magnetic resonance imaging (t-fMRI) from the Human Connectome Project database using a sampling scheme similar to a bootstrapping approach. Brain regions were specified by automated anatomical labeling atlas and the brain activity (*z*-statistics) of each brain region was correlated with body mass index (BMI) values. Regions with significant correlation were identified and the brain activity of the identified regions was correlated with emotion-related clinical scores. Hippocampus, amygdala, and inferior temporal gyrus consistently showed significant correlation between brain activity and BMI and only the brain activity in amygdala consistently showed significant negative correlation with fear-affect score. The brain activity in amygdala derived from t-fMRI might be good neuroimaging biomarker for explaining the relationship between obesity and a negative emotional state.

## Introduction

Obesity is defined as a state of excessive accumulation of body fat and it is a worldwide issue affecting billions of people as it might cause negative health problems such as diabetes, hypertension, and stroke^1^. In addition to physical problems, strong links between obesity and emotional states have been demonstrated in previous studies^2–4^. Using a questionnaire, Ozier *et al*. found a strong correlation between obesity and emotion- and stress-induced eating behaviors^2^. Previous studies found that feelings of anger, loneliness, and disgust were highly linked to eating disorders and obesity, and thus the negative emotional states should be managed properly to prevent and treat eating disorders and obesity^3, 4^. These studies indicated that negative emotional states were related to obesity and they emphasized that understanding the cause of psychological problems that affect obesity was necessary^2–4^.

The previous non-neuroimaging studies largely depended on the questionnaires subject to large individual-level variations. Neuroimaging analysis can provide quantitative information of brain function and thus can complement existing non-neuroimaging studies^5–8^. It is shown that emotional states such as fear, anger, and sadness are highly associated with changes in brain activity^5, 6^. Previous studies adopted functional magnetic resonance imaging (fMRI) technique and found altered activations in amygdala, insula, prefrontal cortex, anterior cingulate cortex, and hippocampus during emotional stimuli^5, 6^. Recent obesity-related studies have utilized neuroimaging to detect structural or functional brain alterations in people with obesity compared to people with healthy weight^7, 8^. In these studies, reward-, emotion-, and cognitive control-related brain regions (i.e., orbitofrontal cortex, amygdala, hippocampus, and insula) showed significant functional differences between people with healthy weight and obesity^7, 8^. However, the finding that emotion-related brain regions show differences between people with healthy weight and obesity based on neuroimaging does not imply that the brain activity in those regions is directly related to emotional states. Most existing obesity related neuroimaging studies aimed to find brain regions that had different brain activity between people with healthy weight and obesity and they did not link emotional states with regional brain activity which this study aimed to accomplish^7, 8^. As there is scarcity of neuroimaging studies that quantitatively link brain function and emotional states in people with obesity, we did not have a priori hypothesis regarding what brain regions to explore. In this study, we adopted the neuroimaging analysis to reveal associative links between brain activity and emotion scores for certain brain regions selected from a set of regions that were related to obesity.

In this study, we used emotion task fMRI (t-fMRI) and hypothesized that regional changes in brain activity would be linked with emotional states. The emotion task was designed to measure the ability to recognize the visual stimuli of angry or fearful facial expression^9–11^. We first sought to find regional imaging features related to obesity. We then quantitatively linked identified neuroimaging features with negative emotional states measured using the NIH toolbox^12, 13^.

## Results

### Identification of obesity-related regions from brain activity

**Figure 1:**
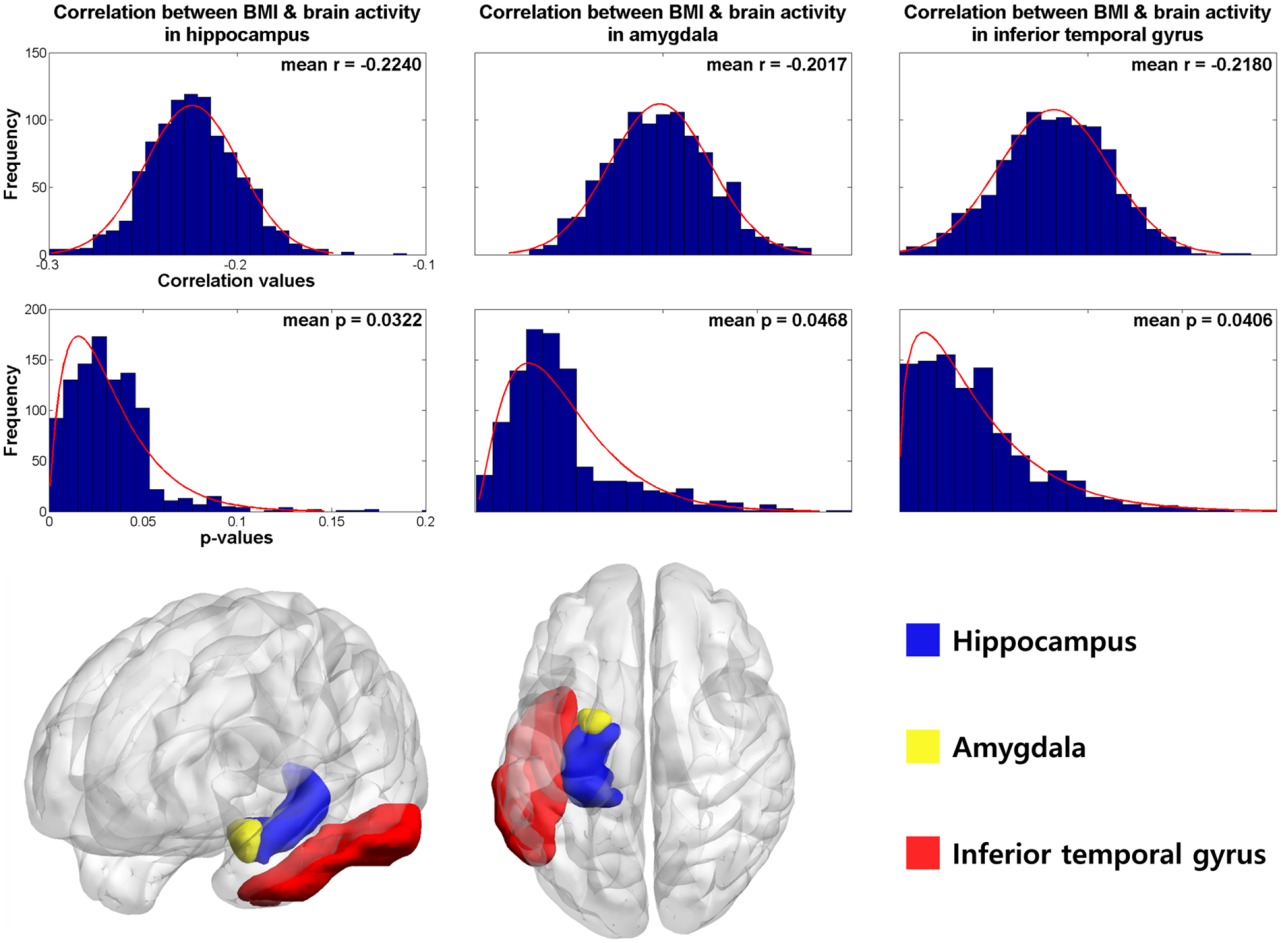
Brain regions that consistently showed significant correlation between brain activity features (*z*-statistics) and BMI for 1,000 times. The histograms of the *r*- and *p*-values were reported in the upper rows and 3D rendered version of the identified regions were shown in the bottom row. The *p*-values were FDR corrected ones.

A total of 196 participants who performed the emotion task^10, 11^ were randomly selected to have matched number of sample size and gender ratio among groups of healthy weight (HW), overweight (OW), class 1 obesity (OB1), and class 2 or 3 obesity (OB23) using a sampling scheme similar to a bootstrapping approach for 1,000 times. Participant-level brain activity during emotion task was measured using FSL software (see Methods section)^14^. The brain activity was quantified using *z*-statistics and they were considered as quantitative imaging features. Brain regions were specified by automated anatomical labeling (AAL) atlas via image registration^15^. The brain regions that consistently showed significant correlation (mean *p* < 0.05, false discovery rate (FDR) corrected) from 1,000 sets of samples were the left hippocampus, amygdala, and inferior temporal gyrus (mean *r* = −0.2240, mean *p* = 0.0322; mean *r* = −0.2017, mean *p* = 0.0468; mean *r* = −0.2180, mean *p* = 0.0406, FDR corrected, respectively) (Fig. 1). The correlation between brain activity and BMI showed negative correlation which implied that the brain activity in people with healthy weight was higher than that in people with obesity.

### Associations between imaging features and emotion-related clinical scores

**Figure 2:**
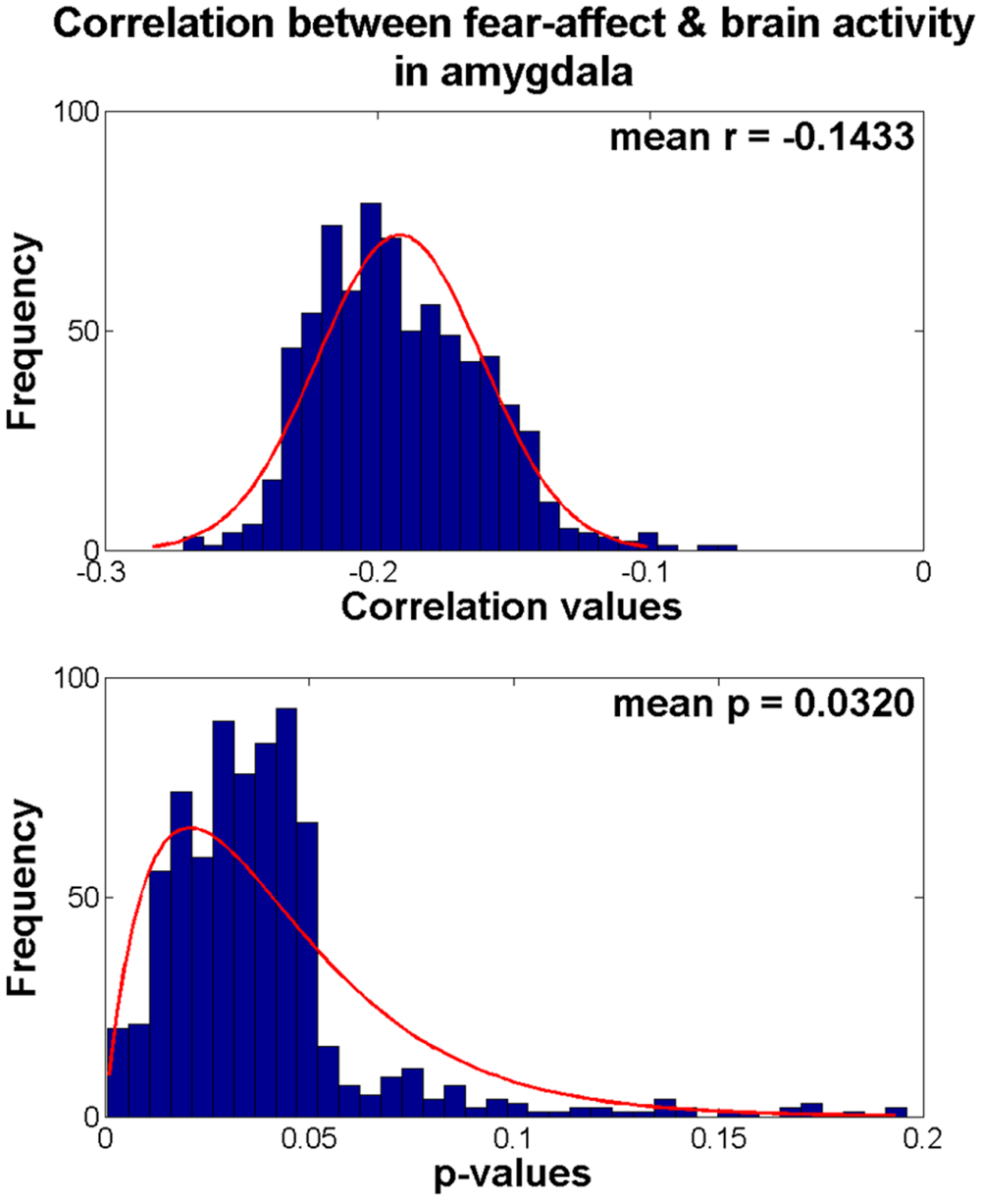
The histogram of the *r*- and *p*-values between the fear-affect score and brain activity features (*z*-statistics) in left amygdala from 1,000 sets of samples.

The brain activity of the identified clusters was quantified using *z*-statistics and they were considered as quantitative imaging features. The brain activity features (*z*-statistics) were stacked across randomly selected participants and they were correlated with emotion-related clinical scores measured using NIH toolbox for 1,000 times (see Methods section)^12, 13^. Only the fear-affect score showed significant correlation with brain activity features in amygdala (mean *r* = −0.1433, mean *p* = 0.0320, Holm-Bonferroni corrected) (Table 1 and Fig. 2). No brain activity features for hippocampus and inferior temporal gyrus showed significant correlation with emotion-related clinical scores. We performed additional correlation analysis between BMI and emotion-related clinical scores to determine if BMI alone explained emotional states, but no emotion-related scores showed significant correlation with BMI (Table 1).

**Table 1:** Correlation between the brain activity features (*z*-statistics) of the identified brain regions and emotion-related clinical scores.

Scores	Left hippocampus		Left amygdala		Left inferior temporal gyrus		BMI	
	*r*	*p*, *corrected*	*r*	*p*, *corrected*	*r*	*p*, *corrected*	*r*	*p*, *corrected*
Anger-affect	−0.0955 (0.0516)	0.4158 (0.3586)	−0.0837 (0.0602)	0.3573 (0.3528)	−0.0753 (0.0618)	0.3814 (0.3838)	0.0430 (0.0332)	0.5569 (0.2399)
Anger-hostility	−0.1012 (0.0513)	0.3552 (0.3386)	−0.0822 (0.0589)	0.3267 (0.3456)	−0.1074 (0.0773)	0.2319 (0.3177)	0.0541 (0.0368)	0.4757 (0.2485)
Anger-physical aggression	0.0328 (0.0437)	0.7897 (0.3356)	−0.0045 (0.0353)	0.6952 (0.4256)	0 (0.0373)	0.6859 (0.4387)	0.0074 (0.0337)	0.7145 (0.1965)
***Fear*** **-** ***affect***	−0.1383 (0.0607)	0.1767 (0.2730)	**−** ***0***.***1433*** (***0***.***0874***)	***0***.***0320*** (***0***.***0438***)	−0.1171 (0.0821)	0.1647 (0.2685)	−0.0065 (0.0381)	0.6899 (0.2182)
Fear-somatic arousal	−0.1103 (0.0584)	0.2771 (0.3091)	−0.1486 (0.0934)	0.0614 (0.1361)	−0.1028 (0.0751)	0.2141 (0.3019)	0.0570 (0.0394)	0.4567 (0.2563)
Sadness	−0.1190 (0.0567)	0.3239 (0.3474)	−0.0744 (0.0528)	0.4049 (0.3641)	−0.0930 (0.0675)	0.3229 (0.3683)	−0.0039 (0.0377)	0.6895 (0.2112)

Means and standard deviations of *r*- and *p*-values from 1,000 sets of randomly selected participants are reported. Significant (*p* < 0.05, Holm-Bonferroni corrected) results are in italic bold.

BMI, body mass index.

### Brain activity in identified regions and obesity

We found significant negative correlation between brain activity features in amygdala and fear-affect score. The results indicated that a person with stronger brain activity in amygdala during emotion t-fMRI might feel less fear-affect than a person with weaker brain activity in the same region. We also found negative correlation between brain activity in amygdala and BMI (Fig. 1), which indicated that brain activity in amygdala of people with obesity were lower than that in people with healthy weight. The results suggested that the brain activity was weaker in amygdala in people with obesity, and it might be associated with increased susceptibility to fear.

## Discussion

We explored differences in brain activity across full range of BMI values using emotion t-fMRI. The brain activity in hippocampus, amygdala, and inferior temporal gyrus showed significant correlation with BMI. The *z*-statistics extracted from the identified brain regions were used as imaging features to explain emotion-related clinical scores. The brain activity features (*z*-statistics) were correlated with emotion-related clinical scores and only the features of amygdala showed significant correlation with fear-affect score. The brain activity of hippocampus and inferior temporal gyrus did not show significant correlation with clinical scores.

It is shown that amygdala plays an important role in emotional recognition especially the expression of fear response^9, 16, 17^. A previous study observed increased brain activity in amygdala when fear condition was given and patients with damaged amygdala showed less response to the conditioned fear^18^. Amygdala is also related to obesity as well as emotional processing^9, 19, 20^. Holsen *et al*. found increased activation in amygdala to food stimuli and King *et al*. found association between dysfunction in amygdala and excessive weight gain^19, 20^. Our results demonstrated that only the left, not right, amygdala showed significant correlation with fear-affect score. Amygdala is known to show functional asymmetry between left and right hemispheres. The left amygdala is more engaged in processing of fearful stimuli than right amygdala^21–25^. Breiter *et al*. found significant brain activity changes in left amygdala when an individual watched fearful faces^23^ and Morris *et al*. found increased brain activity in left amygdala when fearful faces were presented compared to happy faces^24, 25^. Our results corroborated previous studies^21–25^. Hippocampus is widely regarded as an important region responsible for cognitive dysfunction and dementia, but recent studies have indicated that structural and functional alterations of hippocampus are highly related to obesity^26–31^. Smaller hippocampal volumes were found in obese adolescents with metabolic syndrome and a strong relationship between midlife obesity and hippocampal atrophy was identified^28, 29^. Previous study demonstrated that dysfunction in hippocampus is highly associated with excessive food intake which might lead weight gain^32^. A genetic study indicated that the mechanism of SIRT1 gene expression, one of the memory-associated genes, in hippocampus was suppressed in people with obesity and it led to impairment in memory^27^. Inferior temporal gyrus is known to be partly related to obesity^33, 34^. In the previous studies, people with obesity showed increased cerebral blood flow in temporal cortex compared to people with healthy weight and significant brain activation was found in inferior temporal gyrus to food stimuli^33, 34^. Previous studies showed hippocampus, amygdala, and inferior temporal gyrus were related to obesity and amygdala was also highly associated with emotional processing^27–29, 35–37^. The adopted stimuli and direction of differential effects were not exactly same as our study, but the results that amygdala was related to both obesity and emotional states were partly consistent with our results.

Our study has a few limitations. First, the number of participants in class 3 obesity was insufficient. Future studies with larger samples in class 3 obesity are necessary. Second, we used only t-fMRI. Multimodal imaging features that can be derived from other complementary imaging modalities such as rs-fMRI and diffusion tensor imaging might provide better information linking neuroimaging findings with emotional scores.

In summary, we identified brain regions that were significantly related to BMI using emotion t-fMRI. Hippocampus, amygdala, and inferior temporal gyrus showed significant correlation with BMI. Only brain activity for amygdala, not hippocampus and inferior temporal gyrus, showed significant correlation with negative emotional state of fear-affect score. Our results might be used as neuroimaging biomarker for future obesity and emotion-related studies.

## Methods

### Subjects and imaging data

The Institutional Review Board (IRB) of Sungkyunkwan University approved this study. Our study was performed in full accordance with local IRB guidelines. Informed consent was obtained from all participants. We obtained T1- and T2-weighted structural MRI and emotion t-fMRI data from the Human Connectome Project (HCP), an openly accessible research database^38^. The HCP team scanned all participants using a Siemens Skyra 3T scanner at Washington University in St. Louis. Imaging parameters of structural MRI were: voxel resolution = 0.7 mm^3^; number of slices = 256; field of view (FOV) = 224 mm; repetition time (TR) = 2,400 ms for T1-weighted data and 3,200 ms for T2-weighted data; and echo time (TE) = 2.14 ms for T1-weighted data and 565 ms for T2-weighted data. Imaging parameters of emotion t-fMRI data were: voxel resolution = 2.0 mm^3^; number of slices = 72; number of volumes = 176; TR = 720 ms; TE = 33.1 ms; and FOV = 208 × 180 mm. Subjects with drug ingestion or attention problem based on the Diagnostic and Statistical Manual IV (DSM-IV) were excluded^39, 40^. Twin subjects and participants with same parents were excluded. The remaining participants were randomly adjusted to have similar number of sample size and gender ratio among the groups of HW, OW, OB1, and OB23 using a sampling scheme similar to a bootstrapping approach for 1,000 times. Each group had approximately 50 participants with equal ratio between males and females. We considered the BMI as a continuous variable but only for adjusting the number of sample size and gender ratio, participants were grouped into four groups of HW, OW, OB1, and OB23. We matched number of samples in each group since having disproportionally more participants in one group leads to biased result of the particular group and hence increase type I error^41^. BMI in the HW group was greater than or equal to 18.5 and less than 25; BMI in the OW group was greater than or equal to 25 and less than 30; BMI in the OB1 group was greater than or equal to 30 and less than 35; BMI in the OB23 group was greater than or equal to 35^42^. Detailed demographic information is in Table 2.

**Table 2:** Demographic data of all participants (means and standard deviations).

Information	Data
Number of participants (HW:OW:OB1:OB23)	141:139:73:46
Gender (M:F)	158:241
Age (years)	28.83 (3.70)
Anger-affect	47.39 (7.93)
Anger-hostility	50.31 (8.37)
Anger-physical aggression	50.99 (8.67)
Fear-affect	49.59 (7.90)
Fear-somatic arousal	51.52 (8.17)
Sadness	45.79 (7.77)

HW, healthy weight; OW, overweight; OB1, class 1 obesity; OB23, class 2 or 3 obesity; M, male; F, female.

### Task paradigm

**Figure 3:**
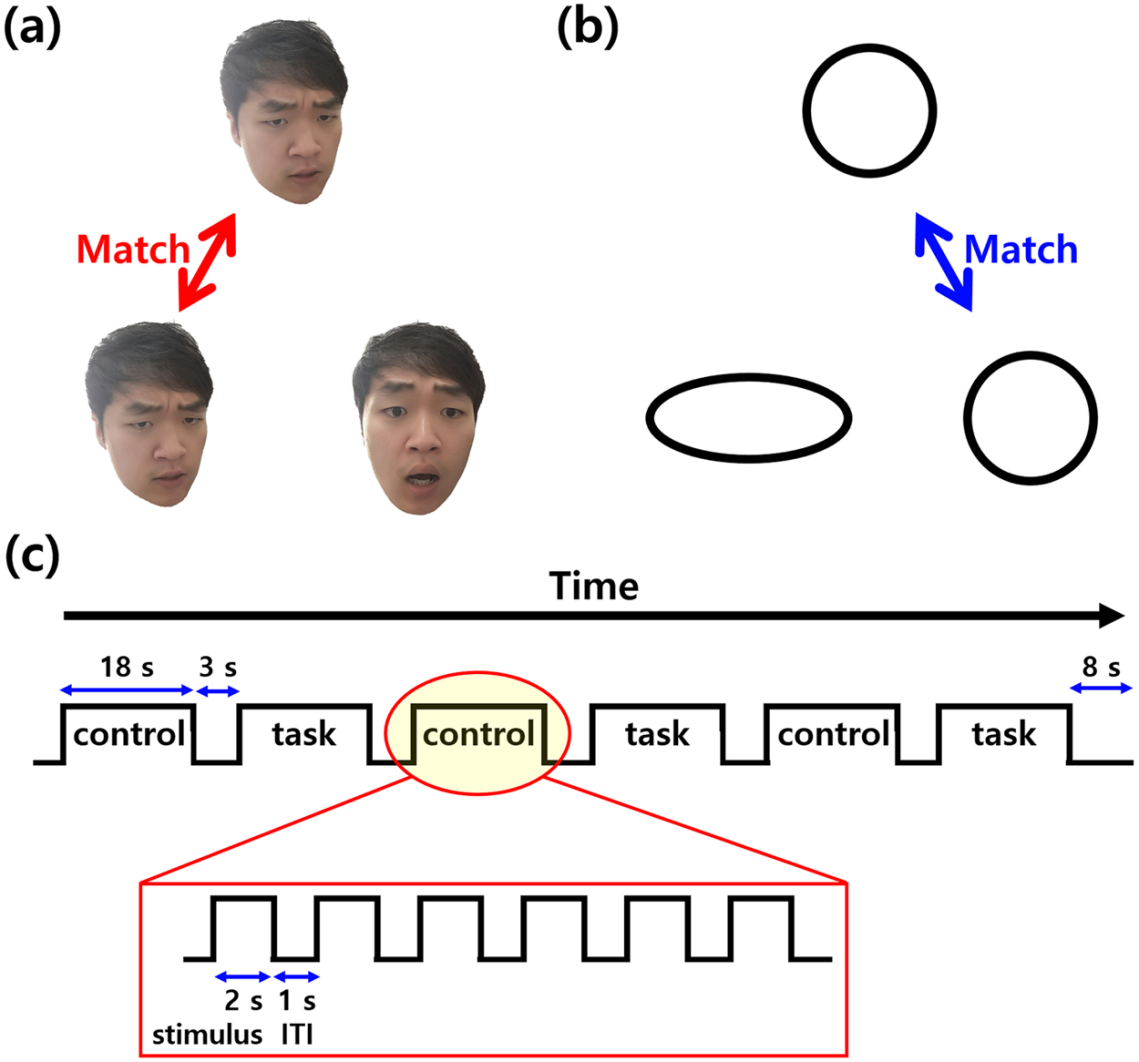
(**a**) Task and (**b**) control states and (**c**) sequences of the emotion task paradigm.

All participants performed the following emotion task^10, 11^. Three faces with either angry or fearful facial expressions were presented on a screen (Fig. 3a). One target face was presented on the top, and two probe faces were presented on the bottom. Participants were asked to select a probe face with the same emotional expression as the target face. The participants saw real human faces as shown in the illustration (Fig. 3a). The control task was the same as the emotion task except that faces were replaced with geometric shapes (Fig. 3b). The task paradigm was designed to match faces with the same emotional expression not to differentiate between emotions. The emotion task paradigm consisted of three tasks and three control blocks that were presented for 21 s. Each block consisted of six trials of 2 s of stimulus (face or shape) and 1 s of inter-task interval (ITI). At the end of all blocks, 8 s of fixation block was presented (Fig. 3c).

### Image preprocessing

We used preprocessed imaging data provided by the HCP database^38, 43^. Imaging data were preprocessed using FSL and FreeSurfer software^14, 44^. T1- and T2-weighted structural MRI data were processed as follows. Gradient nonlinearity and b0 distortions were corrected. T1- and T2-weighted data were registered to each other and averaged. Averaged structural data was aligned onto the Montreal Neurological Institute (MNI) standard space using rigid body transformation. Non-brain tissue was removed by warping the standard MNI brain mask to individual brain data. Magnetic field bias was corrected and registered onto the MNI standard space using nonlinear transformation. Emotion t-fMRI data were processed as follows. Gradient nonlinearity distortion and head motion were corrected. Low-resolution fMRI data were registered onto high-resolution T1-weighted structural data and subsequently onto the MNI standard space. Bias field was corrected, and the skull was removed by applying the standard MNI brain mask to individual participant spaces. Intensity normalization with a mean value of 10,000 was applied to the entire 4D data. Artifacts of head motion, cardiac- and breathing-related contributions, white matter, and scanner-related artifacts were removed using FIX software^45^. We performed the following additional processes. We divided t-fMRI data into several blocks to separate task and control states. Task blocks consisted of fMRI volumes from 6 s of task onset to the first 2 s of task offset to consider delays in hemodynamic response^46^. The HCP database provided data with two distinct phase-encoded directions, “left-to-right” and “right-to-left.” FMRI volumes for task blocks of two phase-encoded data were averaged using the 3dMean function in AFNI software^47^. Volumes of control blocks were formed using the average of both phase-encoding directions. Task blocks and control blocks were concatenated using the fslmerge function in FSL software^14^.

### Task fMRI analysis

Participant-level analysis was conducted using the FEAT framework in FSL software^14^. High-pass filter with cutoff of 200 s and spatial smoothing with full width at half maximum (FWHM) of 4 mm were applied. Two kinds of contrasts were considered. The first was activation of BOLD signals in task compared to control state, and the second was deactivation. A general linear model was constructed to estimate effect sizes as *β* coefficients. Time series of a voxel was the dependent variable, and a design matrix of the start time of task onset and duration was the independent variable. Participant-level contrast of parameter estimate (COPE) was calculated by the linear combination of contrast weight vector and estimated effect size. The *t*-statistics map was computed by dividing COPE with its standard deviation and it was transformed to *z*-statistics map. Brain regions were specified by AAL atlas and the *z*-statistics map of all subjects were used to compute regional brain activity^15^. The regional brain activity was spatial average of activations of a given region. We then correlated regional brain activity with BMI and brain regions that showed significant correlation (*p* < 0.05, FDR corrected) were regarded as significant regions related to obesity.

### Linking imaging features and emotion-related clinical scores

Emotion-related clinical scores were measured using the NIH toolbox^12, 13^. The emotion domain of the toolbox contained four subdomains: negative affect, psychological well-being, stress and self-efficacy, and social relationships^12, 13^. Our study was an exploratory one regarding what negative emotion to focus on and thus we chose to correlate our neuroimaging findings with available negative emotion scores in negative affect subdomain of NIH toolbox with stringent multiple comparison correction. The negative affect subdomain in emotion domain of the NIH toolbox includes anger, fear, and sadness. Anger is the attitudes of hostility and criticism and it includes three sub-components: (1) anger as an emotion (anger-affect), (2) anger as a cynical attitude (anger-hostility), and (3) anger as a behavior (anger-physical aggression). Fear is a symptom of anxiety and perception of threat and it includes two sub-components: (1) psychological emotion of fear and anxiety (fear-affect) and (2) somatic symptoms (fear-somatic arousal). Sadness is a state of low levels of positive affect such as poor mood or depression. Detailed score-related information is reported in Table 2. Identified imaging features of all participants were linearly correlated with emotion-related clinical scores, and the quality of the correlation was assessed using *r*- and *p*-value statistics. *P*-values were corrected using the Holm-Bonferroni method^48^. The behavioral tests of NIH toolbox and emotion task fMRI scan were completed on the same day so that clinical scores of NIH toolbox were reflective of the states that might correlate with the fMRI scan data^10, 38, 49^.
